# Genetic inbreeding load and its individual prediction for milk yield in French dairy sheep

**DOI:** 10.1186/s12711-024-00945-z

**Published:** 2025-01-13

**Authors:** Simona Antonios, Silvia T. Rodríguez-Ramilo, Andres Legarra, Jean-Michel Astruc, Luis Varona, Zulma G. Vitezica

**Affiliations:** 1 GenPhySE, Université de Toulouse, INRAE, ENVT, 31326 Castanet-Tolosan, France; 2 CDCB, 4201 Northview Drive, Bowie, MD 20716 USA; 3 Institut de L’Elevage, 31321 Castanet Tolosan, France; 4 Instituto Agroalimentario de Aragón (IA2), Universidad de Zaragoza, 50013 Saragossa, Spain

## Abstract

**Background:**

The magnitude of inbreeding depression depends on the recessive burden of the individual, which can be traced back to the hidden (recessive) inbreeding load among ancestors. However, these ancestors carry different alleles at potentially deleterious loci and therefore there is individual variability of this inbreeding load. Estimation of the additive genetic value for inbreeding load is possible using a decomposition of inbreeding in partial inbreeding components due to ancestors. Both the magnitude of variation in partial inbreeding components and the additive genetic variance of inbreeding loads are largely unknown. Our study had three objectives. First, based on substitution effect under non-random matings, we showed analytically that inbreeding load of an ancestor can be expressed as an additive genetic effect. Second, we analysed the structure of individual inbreeding by examining the contributions of specific ancestors/founders using the concept of partial inbreeding coefficients in three French dairy sheep populations (Basco-Béarnaise, Manech Tête Noire and Manech Tête Rousse). Third, we included these coefficients in a mixed model as random regression covariates, to predict genetic variance and breeding values of the inbreeding load for milk yield in the same breeds.

**Results:**

Pedigrees included 190,276, 166,028 and 633,655 animals of Basco-Béarnaise, Manech Tête Noire and Manech Tête Rousse, respectively, born between 1985 and 2021. A fraction of 99.1% of the partial inbreeding coefficients were lower than 0.01 in all breeds, meaning that in practice inbreeding occurs in pedigree loops that span several generations backwards. Less than 5% ancestors generate inbreeding, because mating is essentially between unrelated individuals. Inbreeding load estimations involved 658,731, 541,180 and 2,168,454 records of yearly milk yield from 178,123, 151,863 and 596,586 females in Basco-Béarnaise, Manech Tête Noire and Manech Tête Rousse, respectively. Adding the inbreeding load effect to the model improved the fitting (values of the statistic Likelihood Ratio Test between 132 and 383) for milk yield in the three breeds. The inbreeding load variances were equal to 11,804 and 9435 L squared of milk yield for a fully inbred (100%) descendant in Manech Tête Noire and Manech Tête Rousse. In Basco-Béarnaise, the estimate of the inbreeding load variance (11,804) was not significantly different from zero. The correlations between (direct effect) additive genetic and inbreeding load effects were − 0.09, − 0.08 and − 0.12 in Basco-Béarnaise, Manech Tête Noire and Manech Tête Rousse.

**Conclusions:**

The decomposition of inbreeding in partial coefficients in these populations shows that inbreeding is mostly due to several small contributions of ancestors (lower than 0.001) going back several generations (5 to 7 generations), which is according to the policy of avoiding close matings. There is variation of inbreeding load among animals, although its magnitude does not seem enough to warrant selection based on this criterion.

**Supplementary Information:**

The online version contains supplementary material available at 10.1186/s12711-024-00945-z.

## Background

Inbreeding load is the fraction of the mutation load that is due to hidden recessive alleles in heterozygous state. This load, when exposed by inbreeding, is responsible for inbreeding depression, the decrease in performance and fitness in inbred individuals [1].

Inbreeding depression is thought to be due to the presence of recessive alleles in populations or from the reduction of heterozygous loci under overdominance. Usually, inbreeding depression is expected to be larger for fitness traits (e.g. fertility) than in traits less related to fitness (e.g. milk yield). However, there is evidence that inbreeding depression can occur in any trait [2]. For fitness traits, inbreeding depression is mainly endorsed to recessive deleterious mutations. However, for traits under directional selection such as milk yield (selection to increase the mean), dominance deviation effects are on average favourable; and inbreeding depression is due to the reduced expression of dominance effects by an increase in homozygosity [2].

In livestock, inbreeding load can vary among founders, particularly if the founder families were exposed to different selection pressures on deleterious alleles [3]. Inbreeding load of individuals can be predicted in the same manner that we do for additive genetic values based on linear models [4, 5]. However, previous authors [4–6] have never expressed inbreeding load in terms of simple locus effects, e.g. as a substitution effect. In fact, the inbreeding load of individuals is a heritable additive trait, proportional to the gene count of recessive alleles (as it will be shown below), and this trait is only expressed when inbreeding occurs in the descendants [5]. Further, inbreeding load can have a favorable or unfavorable effect on the studied trait (e.g. milk yield) [6]. For instance, one could in principle find out if different individuals carry different inbreeding loads by producing e.g. equally inbred descendance (say mating sires to their daughters) and comparing descendants across sires. In complex pedigrees this becomes more complicated because each individual possesses parts of inbreeding coming potentially from different ancestors. The inbreeding partitioning in fractions attributed to each ancestor can be computed using pedigree, using the Mendelian decomposition of inbreeding, which traces back the specific ancestral paths through which the identical by descent (IBD) alleles are inherited. Using these fractions, a linear model can predict the inbreeding load of the individuals [4, 5] in the same manner that we do for additive genetic values.

There are three objectives of this work. First, we showed that the inbreeding load can be expressed as a genetic additive effect based on substitution effect under non-random matings. Second, we analysed the structure of individual inbreeding by examining the contributions of specific ancestors/founders using the concept of partial inbreeding coefficients in 3 French dairy sheep breeds: Basco-Béarnaise (BB), Manech Tête Noire (MTN) and Manech Tête Rousse (MTR). Third, we used these partial inbreeding coefficients as covariates in a random regression mixed model to estimate genetic variance and breeding values of the inbreeding load for milk yield in the three breeds.

## Theoretical framework of the single-locus inbreeding load concept

Under the assumption of random mating, the substitution effect of a gene is the regression of genotypic values on gene content. However, if mating is non-random, the substitution effect ($\alpha$) is defined (page 347 in Kempthorne [7], Eq. 10 in Falconer [8] and Eq. 4.22 in Lynch and Walsh [9]) as

1 $$\alpha =\frac{e}{(1+F)},$$

where $e$ is the average excess, and $F$ is the inbreeding coefficient which is the reduction of heterozygote frequencies relative to those expected in random mating. The average excess is equal to $e=a(1+F)+d\left(q-p\right)(1-F)$, where $a$ and $d$ are the additive and dominant biological effects, and $p$ and $q$ are the allele frequencies. Substituting $e$ in Eq. (1), the substitution effect in a non-random mating population can be written as.

$$\alpha =a+d\left(q-p\right)\left(\frac{1-F}{1+F}\right).$$

Looking at the term involving $F$, we can write.

$$\left(\frac{1-F}{1+F}\right)=\left(\frac{1+F-2F}{1+F}\right)=1-2\frac{F}{1+F}.$$

So, we can split $\alpha$ in two components, one if there is no inbreeding.

$${\alpha }_{noF}=a+\left(q-p\right)d,$$

And another one, that involves inbreeding.

$${\alpha }_{F}=-2\frac{F}{1+F}\left(q-p\right)d.$$

If $F=0$ then ${\alpha }_{F}=0$ and we get the usual expression for $\alpha =a+d\left(q-p\right)$. If $F=1$ then ${\alpha }_{F}=-2\frac{F}{1+F}\left(q-p\right)d=-2\frac{1}{2}\left(q-p\right)d=-\left(q-p\right)d$*,* in which case we get $\alpha ={\alpha }_{noF}+{\alpha }_{F}=a$. This make sense because if $F=1$ there are only homozygotes.

The inbreeding load ($i$) for an individual is therefore the centered ($\mu =2p$) gene content times the substitution effect with inbreeding, ${\alpha }_{F}$, as follows:

$${i}_{{A}_{1}{A}_{1}}=\left(2-2p\right){\alpha }_{F}=\left(2-2p\right)\left(-2\frac{F}{1+F}\left(q-p\right)d\right)=-4\frac{F}{1+F}q\left(q-p\right)d,$$

$${i}_{{A}_{1}{A}_{2}}=\left(1-2p\right){\alpha }_{F}=\left(1-2p\right)\left(-2\frac{F}{1+F}\left(q-p\right)d\right)=-2\frac{F}{1+F}{\left(q-p\right)}^{2}d,$$

$${i}_{{A}_{2}{A}_{2}}=\left(-2p\right){\alpha }_{F}=\left(-2p\right)\left(-2\frac{F}{1+F}\left(q-p\right)d\right)=4\frac{F}{1+F}p\left(q-p\right)d.$$

Note that the inbreeding load is related to the analyzed trait (e.g. milk yield) and the amount of inbreeding load will depend on the direction of dominance for loci affecting the trait.

At a single locus, the genetic variance due to ${\alpha }_{F}$ is.

$${\sigma }_{i}^{2}=2pq{\alpha }_{F}^{2}=8pq\frac{{F}^{2}}{{\left(1+F\right)}^{2}}{\left(q-p\right)}^{2}{d}^{2}.$$

Note that this variance is expected to be very small, because $\frac{{F}^{2}}{{\left(1+F\right)}^{2}}\approx {F}^{2}$ which is usually very small.

The variance due to ${\alpha }_{noF}$ is the usual expression.

$${\sigma }_{u}^{2}=2pq{a}^{2}+2pq{\left(q-p\right)}^{2}{d}^{2}.$$

In addition, the covariance between $i$ and $u$ (the last is the usual BV in a non-inbred population) is.

$${\sigma }_{u,i}=-4pq\frac{F}{1+F}{\left(q-p\right)}^{2}{d}^{2}.$$

Note that the correlation between the breeding value of the trait and its inbreeding load is always negative. Looking at the magnitude of the squared correlation.

$$\frac{1}{{r}_{(u,i)}^{2}} =\frac{\left(8pq\frac{{F}^{2}}{{\left(1+F\right)}^{2}}{\left(q-p\right)}^{2}{d}^{2}\right)\left(2pq{a}^{2}+2pq{\left(q-p\right)}^{2}{d}^{2}\right)}{16{p}^{2}{q}^{2}{\left(\frac{F}{1+F}\right)}^{2}{\left(q-p\right)}^{4}{d}^{4}}=\frac{\left(2pq{a}^{2}+2pq{\left(q-p\right)}^{2}{d}^{2}\right)}{2pq{\left(q-p\right)}^{2}{d}^{2}},$$

Or in other words.

$${r}_{(u,i)}^{2}=\frac{2pq{\left(q-p\right)}^{2}{d}^{2}}{\left(2pq{a}^{2}+2pq{\left(q-p\right)}^{2}{d}^{2}\right)},$$

The squared correlation is simply the fraction due to dominance gene action ($2pq{\left(q-p\right)}^{2}{d}^{2}$) of the total additive variance in the non-inbred population ($2pq{\alpha}^{2}=2pq{a}^{2}+2pq{\left(q-p\right)}^{2}{d}^{2}$).

## Methods

Data for this study were extracted from the French national dairy sheep database. Animal care and use committee approval was not necessary for this study because the data were obtained from an existing database.

### Phenotypic and pedigree data

Dairy sheep selection schemes have clearly defined and consensual selection objectives that have been updated periodically. Depending on the breed, the breeding objectives include milk yield, fat and protein yields, fat and protein contents, somatic cell score, and udder morphology [10]. All these traits are recorded on farm. A total of 658,731, 541,180 and 2,168,454 records of milk yield from 178,123, 151,863 and 596,586 females of BB, MTN and MTR, respectively, were included. Milk recording is performed according to the International Committee for Animal Recording (ICAR). Average milk yields (± SD) were 193.00 (± 76.25) liters, 144.31 (± 60.25) liters and 197.52 (± 83.66) liters, in BB, MTN and MTR respectively. Pedigrees included 190,276 (186,581 females and 3695 males in BB,), 166,028 (162,584 females and 3444 males in MTN) and 633,655 (622,425 females and 11,230 males in MTR) animals born between 1985 and 2021. By 1985 all breeds had ongoing routine pedigree and milk yield recordings. To assess pedigree completeness, the number of equivalent complete generations was computed using PEDIG software [11]. In all breeds, inbreeding is managed through (i) avoiding mating between individuals with common grandparents and (ii) trying to keep balanced numbers of rams within family of paternal grand-sires at each step of selection.

### Mendelian decomposition of inbreeding

Based on pedigree data, $F$ can be decomposed into coefficients attributed to specific founders known as partial inbreeding coefficients. The inbreeding coefficient of individual $j$ (${F}_{j}$) can be decomposed in a sum of partial inbreeding coefficients each due to an ancestor $k$, e.g. ${F}_{j\left(k\right)}$ where $k$ is an ancestor of $j$. Thus ${F}_{j}=\sum_{k \in ancestors(j)}{F}_{j\left(k\right)}$. Note that most ancestors do not generate partial inbreeding; only those ancestors common to both sides of the pedigree (mother and father of $j$) have ${F}_{j\left(k\right)}\ne 0$.

The partial inbreeding coefficient for animal $j$ attributed to ancestor $k$, (${F}_{j(k)}$), combines the probability that $j$ inherits both maternal and paternal alleles from ancestor $k$ [4, 5, 12] and a Mendelian sampling term which is related to the within-family variation (in other words, the originality of ancestor $k$ from the average of its parents). This decomposition of inbreeding splits inbreeding among founders and the Mendelian sampling of the non-founders [13]. To calculate partial inbreeding coefficients attributed to an animal $j$, we used the approach proposed by García-Cortés et al. [12] which modified the conventional tabular method with a set of recursively formulas. The method operates recursively over ${A}_{(j,k)}=\frac{1}{2}\left({A}_{\left(j,sire\right)}+{A}_{\left(j,dam\right)}\right) +{\phi }_{jk}$, where ${A}_{(j,k)}$ is the additive genetic relationship between individuals $j$ and $k$ (or two times the coancestry between those two individuals), and ${\phi }_{jk}$ is the Mendelian sampling variation and it is related to within-family variation. For a given matrix ${\varvec{\upphi}}=\{{\phi }_{jk}\}$, ${\varvec{\upphi}}=0$ except at the element ${\phi }_{jj}$ of the diagonal that are handled as one of the following options: (1) when both parents are known, we use ${\phi }_{jj}=\frac{1}{4}(1-{F}_{s})+\frac{1}{4}(1-{F}_{d})$ where ${F}_{s}$ (${F}_{d}$) is the inbreeding coefficient of the sire (or dam); (2) when only one parent ($l=s,d$) is known, we use ${\phi }_{jj}=\frac{1}{2}+ \frac{1}{4}\left(1-{F}_{l}\right)$ and (3) when both parents are unknown we use ${\phi }_{jj}=1$. In this method, each individual in the population is seen as a partial founder, such that its Mendelian sampling term contributes to the genetic variability of the population. The term $\phi$ includes the Mendelian sampling variability and the ignorance about the parents [12].

The partial inbreeding coefficients were calculated using a Fortran program available at https://github.com/alegarra/getPartialInbreeding. Partial inbreeding coefficients from the Mendelian decomposition of inbreeding were used later in mixed models for the genetic analysis of milk yield.

### Models

The partial inbreeding coefficients were included in a mixed model as random regression covariates, to predict genetic variance and breeding values of the inbreeding load for milk yield. The effects: flock-year-parity where parity has three classes (1, 2, 3 and more), the age at lambing within year and parity, the period of lambing within year and parity and the lambing-first test-day interval within year and parity, were included in the model as fixed effects. The model including the inbreeding load can be written as

2 $$\mathbf{y}= \mathbf{X}{\varvec{\upbeta}}+\mathbf{f}b+{\mathbf{Z}}_{\text{u}}\mathbf{u}+{\mathbf{Z}}_{\text{u}}\mathbf{K}\mathbf{i}+{\mathbf{Z}}_{\text{p}}\mathbf{p}+\mathbf{e}$$

where $\mathbf{y}$ is the vector of phenotypic records (milk yield), ${\varvec{\upbeta}}$ is the vector of fixed effects, $b$ is the overall inbreeding depression parameter per unit of inbreeding and the covariate $\mathbf{f}$ is the vector of total inbreeding coefficients. The vectors of genetic effects, $\mathbf{u}$ and $\mathbf{i}$, are the additive genetic effect and the inbreeding load effects for milk yield, respectively; such as $$\left( {\begin{array}{*{20}c} {\mathbf{u}} \\ {\mathbf{i}} \\ \end{array} } \right)\sim N\left( {\begin{array}{*{20}c} 0 \\ 0 \\ \end{array} ,{\mathbf{G}} \otimes {\mathbf{A}}} \right)$$; where $$\mathbf{G}=\left[\begin{array}{cc}{\sigma }_{u}^{2}& {\sigma }_{u,i}\\ {\sigma }_{u,i}& {\sigma }_{i}^{2}\end{array}\right]$$; $\mathbf{A}$ is the additive genetic relationship matrix and ${\sigma }_{u,i}$ is the covariance between the additive genetic and the inbreeding load effects. The genetic correlation between the breeding value of milk yield and its inbreeding load was computed as ${r}_{\left(u,i\right)}=\frac{{\sigma }_{u,i}}{\sqrt{{\sigma }_{u}^{2}{\sigma }_{i}^{2}}}$ [6]. The models also included a random permanent effect for each animal ($\mathbf{p}\sim N$(0, $\mathbf{I}{\sigma }_{p}^{2}$)) and the residual ($\mathbf{e}\sim N$(0, $\mathbf{I}{\sigma }_{e}^{2}$)). The incidence matrices $\mathbf{X}$, ${\mathbf{Z}}_{\text{u}}$, and ${\mathbf{Z}}_{\text{p}}$ relate records to fixed effects, and additive genetic and permanent environmental effects, respectively. The matrix $\mathbf{K}$ is a lower triangular matrix, $\mathbf{K}=\mathbf{T}(\mathbf{I}-\mathbf{P})$, where $\mathbf{T}$ contains the partial inbreeding coefficients of all individuals, $\mathbf{I}$ is the identity matrix and the product ${\mathbf{Z}}_{\text{u}}\mathbf{K}$ links the phenotypes of animals in records to their ancestors causing inbreeding. The matrix $\mathbf{P}$ has 0 in its diagonal and its elements 0.5 connect an individual with its sire and dam [5]. An example of how to set up this model is in https://github.com/alegarra/getPartialInbreeding. In this model, the individual recessive burden due to inbreeding in the individual expressing the trait is reformulated using matrix $\mathbf{K}$ into a linear model that uses the inbreeding load of its ancestors, and this is possible because the individual pedigree inbreeding can be decomposed as a sum over ancestors of partial inbreeding coefficients. Thus, the individual recessive burden due to inbreeding in the individual can be modelled as the inbreeding load, an indirect additive genetic effect of some ancestors. This is vaguely similar to models with maternal effects, in which the calf is affected by the maternal ability.

The full model (FM) in Eq. (1) was compared to a model without the inbreeding load called the reduced model (RM): $\mathbf{y}= \mathbf{X}{\varvec{\upbeta}}+\mathbf{f}b+{\mathbf{Z}}_{\text{u}}\mathbf{u}+{\mathbf{Z}}_{\text{p}}\mathbf{p}+\mathbf{e}$. (Co)variance components were estimated using restricted maximum likelihood (REML) for FM and RM. The superiority of the FM over RM was tested by a likelihood ratio test, which was calculated as -2ln(likelihood for RM) + 2ln(likelihood for FM). The likelihood ratio follows a mixture of χ^2^-distributions with 0 and 1 degree of freedom [14].

The matrix $\mathbf{K}$ was computed using a program in Julia available at https://github.com/alegarra/getPartialInbreeding. To avoid computational problems per numerical over/underflows, we included only values of the $\mathbf{K}$ matrix with absolute values higher than 0.01.

Inbreeding $\mathbf{f}$ was calculated with the inbupgf90 program [15] available at http://nce.ads.uga.edu/wiki/doku.php?id=readme.inbupgf90 (although it could equally have been computed summing partial inbreeding coefficients per individual). Programs of the BLUPF90 + family [16] were used to estimate variance components and are available at http://nce.ads.uga.edu/wiki/doku.php.

## Results

### Inbreeding and its Mendelian decomposition

Pedigree-based inbreeding coefficients for each breed are shown in Table 1. Low inbreeding coefficients were estimated for the whole population (less than 1%). Our inbreeding estimates agreed with values obtained in other dairy sheep breeds: Latxa Cara Negra from Euskadi (0.018), Latxa Cara Rubia (0.016), and Latxa Cara Negra from Navarre (0.018) [17]. Among inbred animals, inbreeding coefficients were higher (~ 0.03) and agreed with estimates obtained on genotyped animals of the same breeds [18]. Few animals (less than 1%) presented inbreeding coefficients greater than 0.10 (Table 1).

**Table 1:** Descriptive statistics for inbreeding in the three breeds

Breed	Inbred animals (%)	Fraction (%) of inbred animals with ${\varvec{F}}<0.05$	Fraction (%) of inbred animals with ${\varvec{F}}>0.1$	Average ${\varvec{F}}$ among inbred animals	Average ${\varvec{F}}$ in the whole population
BB	41	85	0.6	0.032	0.013
MTN	24	88	0.9	0.030	0.007
MTR	56	96	0.5	0.025	0.014

BB: Basco-Béarnaise; MTN: Manech Tête Noire; MTR: Manech Tête Rousse. *F*: coefficient of inbreeding

The partial inbreeding coefficients from the Mendelian decomposition of inbreeding are presented in Table 2. A total of 9,775,475, 2,235,928 and 75,119,288 coefficients were generated, belonging to 3855, 3124 and 12,344 ancestors (917, 828 and 2716 sires and 2938, 2296, 9628 dams) in the BB, MTN and MTR breeds, respectively. Note that these are only some of the ancestors, i.e. most ancestors do not generate inbreeding. The total number of ancestors in the three breeds was actually 91,476, 72,467 and 308,848 individuals in BB, MTN and MTR, respectively.

**Table 2:** Distribution of partial inbreeding coefficients in the three breeds

Breed	Nb of coefficients	Average (SD)	Max	Nb of Ancestors involved*
BB	9,775,475	2.4 × 10^−4^ (9.9 × 10^−4^)	0.25	3855
MTN	2,235,928	5.5 × 10^−4^ (2.1 × 10^−3^)	0.25	3124
MTR	75,119,288	1.2 × 10^−4^ (7.2 × 10^−4^)	0.25	12,344

Nb: Number; SD: Standard Deviation; Max: Maximum. BB: Basco-Béarnaise; MTN: Manech Tête Noire; MTR: Manech Tête Rousse. *Ancestors involved: ancestors that generate partial inbreeding coefficients

Note that if the ancestor is distant, the partial inbreeding coefficient is small and if the ancestor is close, the partial coefficient is large. Very few individuals had partial inbreeding coefficients of 0.25: only 4 animals in BB, 7 animals in MTN and 32 animals in MTR. In BB, the partial inbreeding coefficient of 0.25 was due to a relationship where the animal was the result of a mother and son mating. All these high values are old (< 1995) and precede the current organization of artificial insemination and matings in selection schemes.

**Fig. 1:**
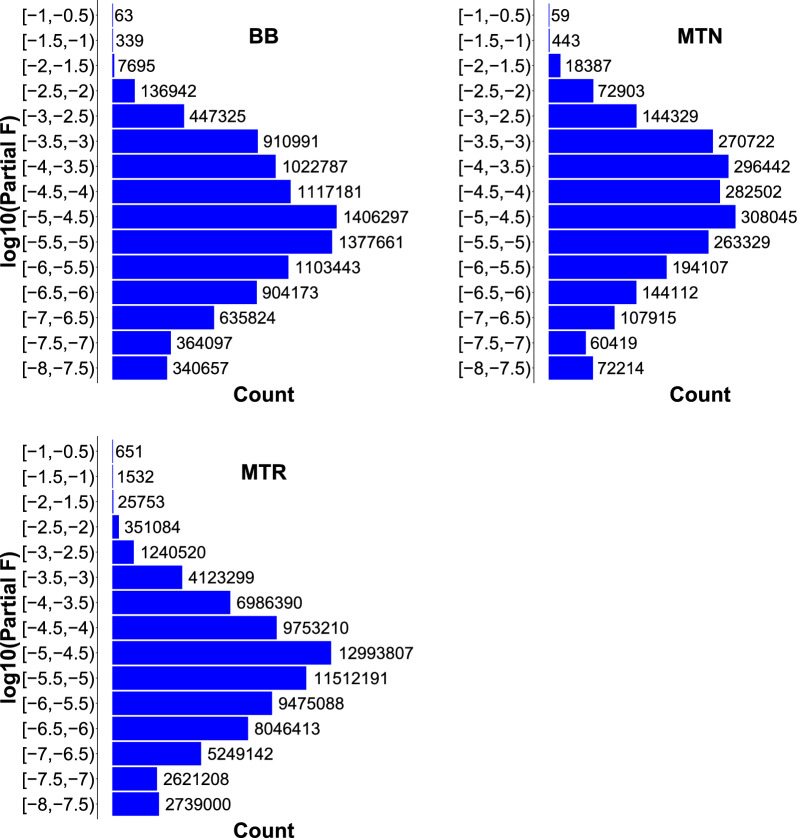
Distribution of the log10 of the partial inbreeding coefficients for animals that generate inbreeding. BB, Basco-Béarnaise; MTN, Manech Tête Noire; MTR, Manech Tête Rousse; Partial F, partial inbreeding coefficient

Figure 1 shows the distribution of the log10 of the partial inbreeding coefficients. Most of the partial inbreeding coefficients, 97.7% and 95.8%, were lower than 0.01 (− 2 in the log10 scale) and 0.001 (− 3 in the log10 scale) on average in the three breeds. Partial inbreeding coefficients greater than 0.01 (− 2 in the log10 scale in Fig. 1) were generated by 732, 519 and 2380 ancestors in BB, MTN and MTR, respectively. Among these ancestors, 13%, 22% and 12% of them were founders in BB, MTN, MTR, respectively. Among all the ancestors that generated inbreeding (Table 2), 20%, 25% and 17% of them were founders in BB, MTN and MTR, respectively.

**Fig. 2:**
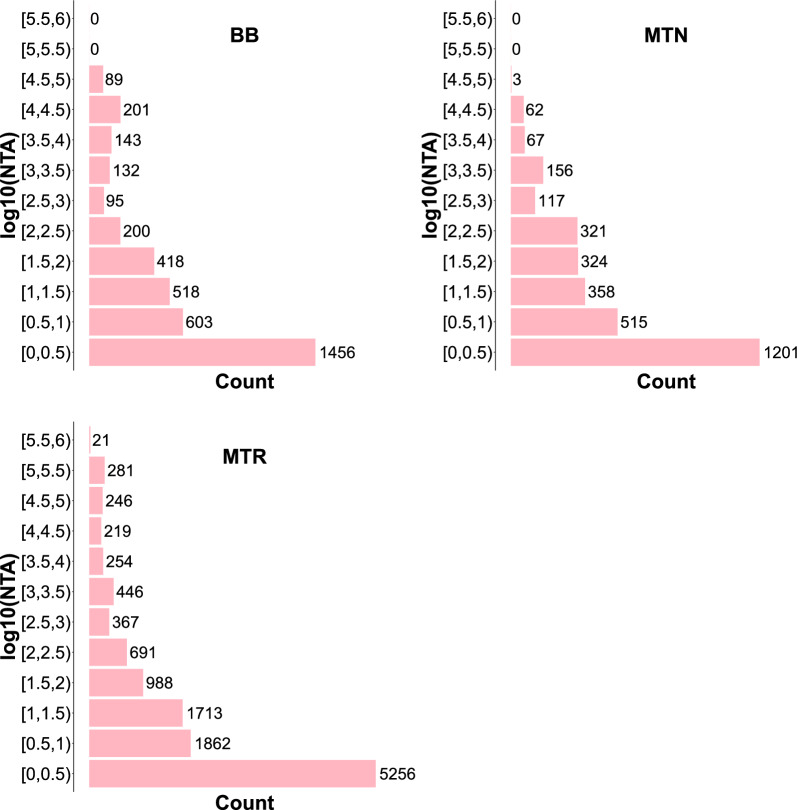
Distribution of the log10 of the number of times that an animal is ancestor and generates inbreeding (NTA). BB, Basco-Béarnaise; MTN, Manech Tête Noire; MTR, Manech Tête Rousse

Figure 2 shows the number of times an animal appeared as an ancestor generating inbreeding (${F}_{j(k)}>0$). Most animals appear a few times, i.e. they generate inbreeding in a small number of descendants, and few animals do generate inbreeding in many descendants. This is true for each of the three breeds. For instance, there were 1456 animals in BB, 1201 animals in MTN and 5256 animals in MTR which were present less than ${10}^{0.5}\approx 3$ times as ancestors generating inbreeding. The number of animals which contributed to partial inbreeding more than ${10}^{4.5}\approx \text{32,000}$ times was only 89, 3 and 548 ancestors in BB, MTN and MTR respectively. It is on this kind of animals (animals whose inbreeding load is expressed across several descendants) that accurate estimation of variance components relies. The number of equivalent complete generations was computed for the three breeds and it was equal to 7.04, 6.18 and 7.82 for BB, MTN and MTR breeds, respectively.

### Genetic analysis

Genetic parameters obtained using models with (FM) and without the inbreeding load (RM) are presented in Table 3. The estimate of inbreeding load genetic variance (${\sigma }_{i}^{2}$) was very inaccurate with large standard error in BB. On the contrary, ${\sigma }_{i}^{2}$ was highly different from zero for MTN and MTR. When we look at the likelihood ratio test** (**LRT) (Table 4) the perspective is somewhat different, and the null hypothesis of null inbreeding load genetic variance was rejected for all breeds. The obtained estimates of ${\sigma }_{i}^{2}$ clearly indicate variability of inbreeding load for milk yield among ancestor families in MTN and MTR breeds. For BB, it is not possible to definitely affirm that there is sizeable genetic variance of inbreeding load for milk yield.

**Table 3:** Parameter estimates for milk yield (liters) obtained using the two models RM and FM (SE)

**Breed**	**Model**	${{\varvec{\sigma}}}_{{\varvec{u}}}^{2}$	${{\varvec{\sigma}}}_{{\varvec{i}}}^{2}$	${{\varvec{\sigma}}}_{{\varvec{u}},{\varvec{i}}}$	${{\varvec{r}}}_{\left({\varvec{u}},{\varvec{i}}\right)}$	${{\varvec{\sigma}}}_{{\varvec{p}}}^{2}$	${{\varvec{\sigma}}}_{{\varvec{e}}}^{2}$	${\varvec{b}}$
BB	RM	847.7 (10.9)	–	–	–	417.3 (7.2)	1325.1 (2.7)	− 111.8 (8.2)
	FM	847.9 (10.9)	11,804.0 (7356.2)	− 289.0 (514.8)	− 0.09 (0.3)	417.0 (7.2)	1325.1 (2.7)	− 109.0 (11.4)
MTN	RM	681.1 (9.5)	–	–	–	363.1 (6.8)	958.5 (2.2)	− 95.9 (9.3)
	FM	678.3 (9.5)	9434.7 (4089.5)	− 192.5 (436.5)	− 0.08 (0.2)	364.9 (6.8)	958.5 (2.2)	− 73.0 (13.9)
MTR	RM	1206.4 (8.0)	–	–	–	513.0 (4.8)	1492.9 (1.7)	− 70.9 (4.5)
	FM	1205.7 (8.0)	12,923.0 (3627.1)	− 460.1 (269.6)	− 0.12 (0.1)	513.2 (4.8)	1492.9 (1.7)	− 50.6 (7.5)

BB: Basco-Béarnaise; MTN: Manech Tête Noire; MTR: Manech Tête Rousse. RM: reduced model (without inbreeding load effect); FM: full model (with inbreeding load effect). ${\sigma }_{u}^{2}$: additive genetic variance; ${\sigma }_{i}^{2}$: inbreeding load variance; ${\sigma }_{u,i}$: covariance between additive genetic and inbreeding load effects; ${r}_{\left(u,i\right)}$: correlation between additive genetic and inbreeding load effects; ${\sigma }_{p}^{2}$: permanent environment variance; ${\sigma }_{e}^{2}$: residual variance; $b$: inbreeding depression expressed by completely inbred (100%) descendants

**Table 4:** Likelihood ratio test (LRT) of models included inbreeding load (FM) or not (RM)

Breed	− 2 log Likelihood		LRT	
	**FM**	**RM**	${{\varvec{\chi}}}^{2}$	***P*-value**
BB	6,759,011.664	6,759,143.696	132.032	7.4 × 10^–31^
MTN	5,379,562.702	5,379,715.675	152.973	1.9 × 10^–35^
MTR	22,626,973.729	22,627,357.574	383.845	9.1 × 10^–86^

BB: Basco-Béarnaise; MTN: Manech Tête Noire; MTR: Manech Tête Rousse. FM: full model (with inbreeding load effect); RM: reduced model (without inbreeding load effect). ${\chi }^{2}$: chi-square value

The inbreeding load genetic variance was larger than the additive genetic variance. This is largely a scale effect due to the small numbers involved in partial inbreeding coefficients. Note that the model gives individual predictions of the inbreeding load genetic effect. For an individual, this value must be understood as the effect expressed on the phenotype (milk yield) by a completely inbred (100%) descendant, with the inbreeding of this descendant coming from the individual under consideration. We rescaled the ${\sigma }_{i}^{2}$ to a meaningful average value of $F$ of 0.10. Considering this value of $F$, the rescaled inbreeding load variances were 118.04 ($={\sigma }_{i}^{2}{\left(0.10\right)}^{2}=\text{11,804.0}{\left(0.10\right)}^{2}$, Table 3) in BB, 94.35 in MTN and 129.23 in MTR. This rescaled variance corresponds to 4.3%, 4.5% and 4.0% of the phenotypic variance in BB, MTN and MTR, respectively.

From estimates of the genetic parameters, the genetic correlation between additive genetic and inbreeding load effects (${r}_{\left(u,i\right)}$) is negative (as expected), small and with large standard errors in all cases (Table 3). An additional file includes the bivariate plot showing the relationship between additive genetic and inbreeding load effects for the three breeds (see Additional file 1: Figure S1).

Overall inbreeding depression ($b$), based on the total inbreeding coefficients, was detected for milk yield in the three breeds (Table 3). With both models, the estimate of inbreeding depression was on average equal to $\widehat{b}=- 110.4$ liters of milk yield in BB breed. This means that a 10% increase in inbreeding would result in a reduction of 11 L of milk yield in this breed. In MTN and MTR, estimates of inbreeding depression differed between the FM and RM models. When the model included the inbreeding load (FM), a reduction of around 26% or 2.2 L for 10% increase in inbreeding, was observed in the estimates of inbreeding depression $b$ in MTN and MTR. Based on the FM model, overall inbreeding depression rates $b$ for milk yield, expressed as a percentage of the population mean, were 0.56%, 0.51%, and 0.26% for each 0.01 (1%) increase in inbreeding in BB, MTN, and MTR, respectively. These values are comparable to previous findings, where a 0.37% decrease per 1% increase in inbreeding was estimated for milk yield in Holstein [1].

**Fig. 3:**
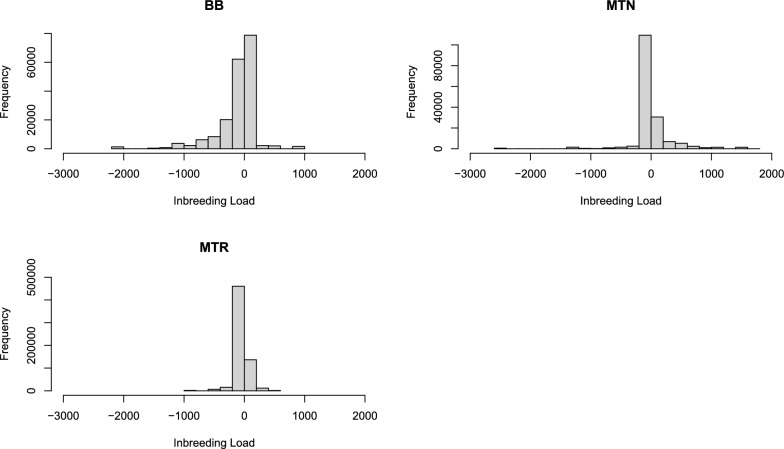
Distribution of the predicted inbreeding load genetic effects in the three breeds (all animals). BB, Basco-Béarnaise; MTN, Manech Tête Noire; MTR, Manech Tête Rousse

The distribution of the predicted inbreeding load was presented in Fig. 3. The averages of the predicted inbreeding loads were − 116.26, 6.42 and − 47.91 for BB, MTN and MTR respectively. Some of the predictions are very high (e.g. higher than 1000) and this may be due to the inaccuracy of the prediction. The proportion of individuals with a positive predicted inbreeding load, higher than zero, was 44%, 24% and 30% in BB, MTN and MTR, respectively. However, this does not include the overall inbreeding depression estimate $-b$. When we subtract this value, 9%, 16% and 11% of the individuals in the three breeds (BB, MTN and MTR respectively) have positive predicted inbreeding, i.e. they would compensate the overall inbreeding depression and even produce a positive inbreeding effect. This indicates an improving in milk yield of their inbred descendants. Let’s take the example of the MTR breed, $\widehat{b}\approx -50$ liters, and consider an animal that has a prediction of inbreeding load of + 60 L. In a hypothetical 100% inbred descendant of this animal, the total inbreeding effect would be $60+\left(-50\right)=10$, i.e., inbreeding load would compensate the negative effect of the overall inbreeding depression yielding + 10 L of increase in the milk yield.

## Discussion

The Mendelian decomposition partitions inbreeding into partial inbreeding coefficients attributed to specific ancestors. Among all ancestors in the pedigrees (91,476, 72,467, 308,848), only 4.2%, 4.3%, 4.0% of them generate partial inbreeding, in BB, MTN and MTR, respectively. Among these ancestors generating inbreeding, only ~ 25% in MTN (and even less in the other 2 breeds) of them were founders (animals whose ancestors are unknown). Thus, inbreeding comes mainly from the Mendelian sampling of non-founders. Most partial inbreeding coefficients (~ 90%) had values lower than 0.001. These results highlight the good management of inbreeding achieved in these breeds through mating plans that avoid mating among cousins (in particular for inseminations) and through husbandry practices (for natural mating, e.g. not using rams from the same farm).

Estimates of additive genetic variance of inbreeding loads were significantly different from zero in milk yield in MTN and MTR, but not in BB. We do not have a clear explanation for this result. The reason is not pedigree length harming the estimation, because BB has a pedigree less deep than MTR (7.04 vs. 7.82 generations) but deeper than MTN (7.04 vs. 6.18 generations). An alternative hypothesis would be removal of variation in inbreeding load due to purging. In the context of inbreeding load, old inbreeding would correspond to low values of partial inbreeding and new inbreeding to high values. If selection for milk yield is strong relative to drift, purging occurs leading to elimination of deleterious alleles, and therefore there would be no variation left. To test the purging, recent and old inbreeding were analysed using Kalinowski’s inbreeding coefficients (${F}_{KAL}$; [19]). However, variance of ${F}_{KAL}$ was very small (0.000077 in BB, 0.000057 for MTN and 0.000037 in MTR) compared with the variance of $F$ (0.0004 in BB, 0.0004 in MTN and 0.0003 in MTR) for the three breeds. Thus, including ${F}_{KAL}$ as a covariate in a mixed model has no power to detect inbreeding depression in these data sets. Our results are in line with Antonios et al. [20], that could not confirm purging in BB. Still, BB has the smallest effective population size of the three breeds (59 based on Runs of Homozygosity, compared to 81 for MTN and 109 for MTR) [18], and this could have led to purging. However, we cannot confirm this hypothesis.

Estimates of the genetic correlation between the additive genetic value and the inbreeding load were negative (as expected from the theory presented above) and low (~ − 0.1). For all the breeds, the correlations were near zero and had a large standard error. Compared to the results obtained by Varona et al. [5], our estimates of this genetic correlation were much closer to zero than their estimate for Pirenaica beef cattle (~ − 0.4). Small and negative genetic correlations were also reported in Brown Swiss dairy cattle for fertility traits [6]. Milk yield is highly selected for in the breeds in this study [10]. The low values of the genetic correlation between inbreeding load and breeding values imply that selection for milk yield will not cause an increase in inbreeding depression in milk yield in inbred animals.

Prediction of inbreeding load of individuals without progeny is possible based on relatives with inbred descendants. Artificial purging based on predicted inbreeding load effects could be performed to reduce the effect of inbreeding depression as suggested by Varona et al. [5] and Martinez-Castillero et al. [6]. Even if this artificial purging is feasible in theory, the magnitude of inbreeding load effects predicted in this study does not seem large enough to warrant selection based on this criterion. The use of artificial purging strategies requires the existence of variability in the inbreeding load among the individuals (our studies confirm that there is) and inbreeding load effects accurately predicted for each individual as we discuss below.

Accurate estimates of inbreeding load effects are an issue. Here we used pedigree information to estimate inbreeding coefficients. In these populations, the pedigree structure is such that estimation of individual inbreeding loads will generally be inaccurate, and this is true for all traits. However, the use of SNP markers to predict inbreeding load could be more accurate [21]. We did not use genomic data because genomic selection was introduced in these dairy sheep breeds in 2016, which means that there are not enough animals genotyped for that purpose and we would need females to be genotyped on a regular basis, which is not the case in our dairy sheep breeds.

Selecting individuals based on predicted inbreeding load for milk yield would basically remove recessive alleles reducing milk yield in homozygote carriers. However, recessive alleles for milk yield may have a pleiotropic effect on fitness traits, and selection to eliminate them may increase or decrease fitness. Currently, options to deal with decreases in fitness due to selection for economical traits include selection for fitness traits such as fertility, or mate allocation strategies to avoid genetic effects [22]. Thus, instead of using the inbreeding load predictions for selection, they could be used to avoid undesirable matings. Still, the magnitude of these effects in this study precludes this strategy. Further research in other species and traits is needed to explore the possible, if any, benefits of these genetic management strategies.

## Conclusions

We present theory that shows that recessive effects can be modelled as an additive trait in the ancestors (called inbreeding load). The inbreeding load additive effect and the regular (in a non-inbred population) additive genetic effect have a negative correlation depending on allele frequencies, inbreeding and biological dominance. There was genetic variance for inbreeding load in the MTN and MTR breeds, but it was not significantly different from zero for BB. As expected, we estimated negative genetic correlations between inbreeding load and breeding values; however, estimates were close to zero in the three sheep breeds. The small magnitude of inbreeding load does not warrant selection based on this criterion.

## Supplementary Information

Additional file 1. Figure S1. Bivariate plot showing the relationship between additive genetic and inbreeding load effects for Basco-Béarnaise (BB), Manech Tête Noire (MTN) and Manech Tête Rousse (MTR) breeds.

## Data Availability

The data that support the findings of this study are available from the breeders, but restrictions apply to the availability of these data, which are not publicly available. Software is available in https://github.com/alegarra/getPartialInbreeding.
