# Supplementary material for: Genetic inbreeding load and its individual prediction for milk yield in French dairy sheep
Source: Genet Sel Evol. 2025 Jan 13;57:1. doi: 10.1186/s12711-024-00945-z (PMC11727507; doi:10.1186/s12711-024-00945-z)
Supplement: Supplementary file 1 — Additional file 1. Figure S1. Bivariate plot showing the relationship between additive genetic and inbreeding load effects for Basco-Béarnaise (BB), Manech Tête Noire (MTN) and Manech Tête Rousse (MTR) breeds. [file 12711_2024_945_MOESM1_ESM.pdf]

Additional file 1

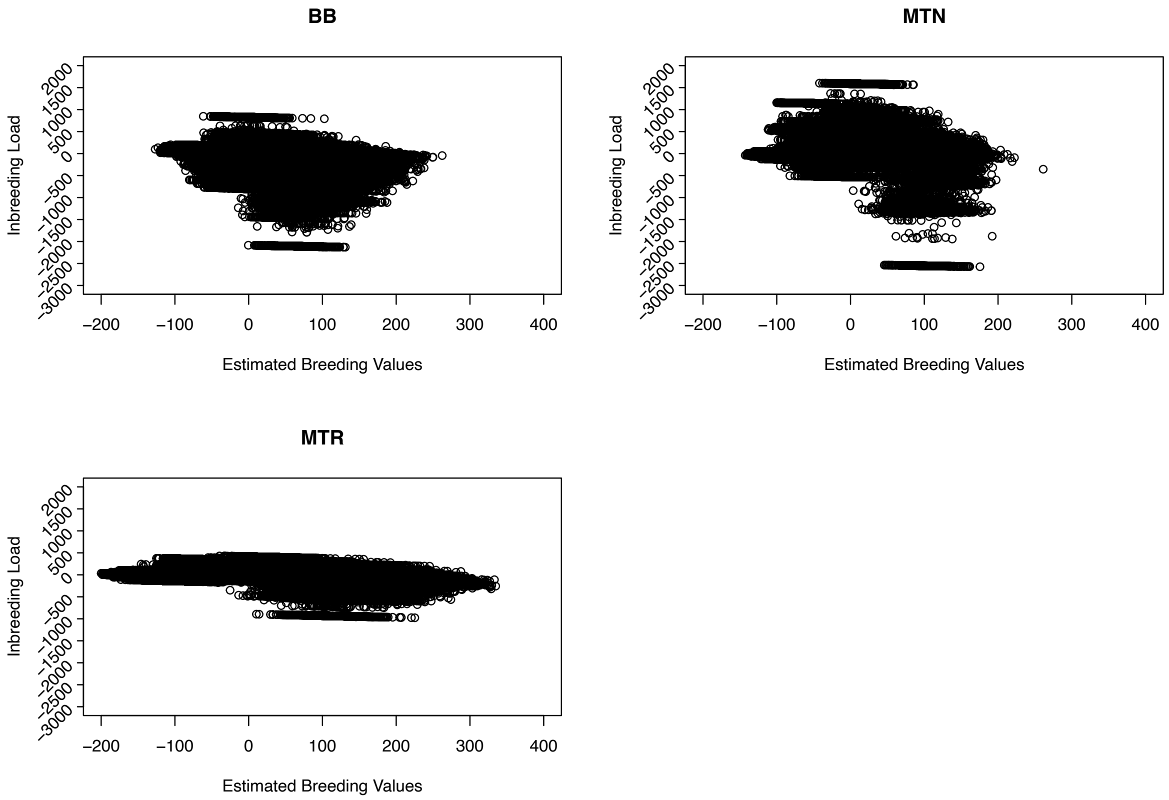

Figure S1. Bivariate plot showing the relationship between additive genetic and inbreeding load effects for Basco-Béarnaise (BB), Manech Tête Noire (MTN) and Manech Tête Rousse (MTR) breeds.
